# On the way to SI traceable primary transfer standards for amount of substance measurements in inorganic chemical analysis

**DOI:** 10.1007/s00216-023-04660-4

**Published:** 2023-04-01

**Authors:** Ralf Matschat, Silke Richter, Jochen Vogl, Heinrich Kipphardt

**Affiliations:** Bundesanstalt für Materialforschung und -prüfung (BAM), Unter den Eichen 87, 12205 Berlin, Germany

**Keywords:** Inorganic chemical analysis, Primary transfer standards (PTSs), Traceability, Classical primary measurement method (CPM), Primary difference measurement method (PDM), Metrology in chemistry

## Abstract

During its 25 years of existence, the Inorganic Analysis Working Group of the Consultative Committee for Amount of Substance: Metrology in Chemistry and Biology (CCQM IAWG) has achieved much in establishing comparability of measurement results. Impressive work has been done on comparison exercises related to real-world problems in fields such as ecology, food, or health. In more recent attempts, measurements and comparisons were focused on calibration solutions which are the basis of most inorganic chemical measurements. This contribution deals with the question of how to achieve full and transparent SI traceability for the values carried by such solutions. Within this framework, the use of classical primary methods (CPMs) is compared to the use of a primary difference method (PDM). PDM is a method with a dual character, namely a metrological method with a primary character, based on the bundling of many measurement methods for individual impurities, which lead to materials with certified content of the main component. As in classical methods, where small corrections for interferences are accepted, in PDM, many small corrections are bundled. In contrast to classical methods, the PDM is universally applicable to all elements in principle. Both approaches can be used to certify the purity (expressed as mass fraction of the main element) of a high-purity material. This is where the metrological need of National Metrology Institutes (NMIs) for analytical methods meet the challenges of analytical methods. In terms of methods, glow discharge mass spectrometry (GMDS) with sufficient uncertainties for sufficiently small impurity contents is particularly noteworthy for the certification of primary transfer standards (PTS), and isotope dilution mass spectrometry (IDMS), which particularly benefits from PTS (back-spikes) with small uncertainties, is particularly noteworthy for the application. The corresponding relative uncertainty which can be achieved using the PDM is very low (< 10^−4^). Acting as PTS, they represent the link between the material aspect of the primary calibration solutions and the immaterial world of the International System of Units (SI). The underlying concepts are discussed, the current status of implementation is summarised, and a roadmap of the necessary future activities in inorganic analytical chemistry is sketched. It has to be noted that smaller measurement uncertainties of the purity of high-purity materials not only have a positive effect on chemical measurements, but also trigger new developments and findings in other disciplines such as thermometry or materials science.

**Graphical Abstract:**

Primary Transfer Standards (PTSs) are the link between the immaterial world of the International System of Units (SI) and the material aspects of the primary calibration solutions.

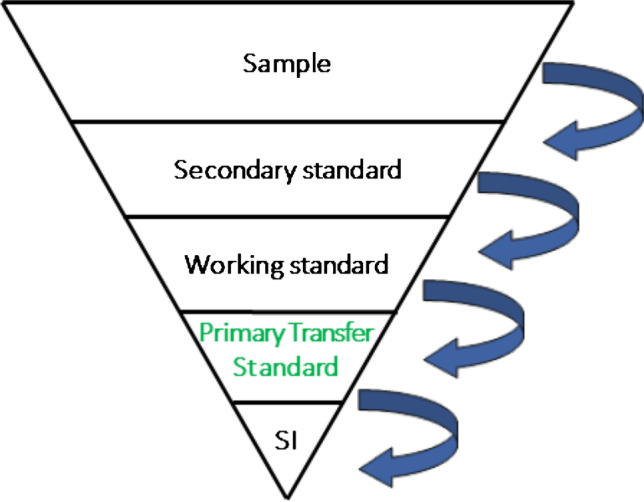

## Introduction

The Consultative Committee for Amount of Substance: Metrology in Chemistry and Biology (CCQM) is one of the consultative committees of the International Committee for Weights and Measures (CIPM) representing the seven base units of the International System of Units (SI). Its main responsibility is “to establish global comparability of measurement results through promoting traceability to the SI” [1–3]. The way forward to achieve this goal is by metrologically linking the results of a chemical measurement to a primary realization of the corresponding SI unit through an unbroken chain of calibrations [4]. In order to avoid traceability chains that are not traced back to the primary standard of a National Metrology Institute (NMI, throughout this paper the mention of NMIs include the Designated Institutes DIs), the responsibility of the NMIs has been confirmed by statutory provisions and, in most countries, by the signing of the State Treaty on the Meter Convention. It is the responsibility of accreditors of the International Laboratory Accreditation Cooperation (ILAC) to verify and require the traceability chains used by accredited laboratories to their primary starting point at their country’s or another country’s national primary standards of the NMI. The formal introduction of metrology in chemistry under the umbrella of the CIPM was not established before 1993 [5]. In contrast to physical measurements, in chemical measurements, the identity of the analyte matters and is part of the measurand definition; in the frame of this work, we focus on inorganic chemical analysis where the elements of the periodic table are of importance.

The initial aim of CCQM was to achieve and to demonstrate comparability of measurement results. In the beginning, urgent problems of the society were addressed in an appropriate timeframe, i.e., in a pragmatic way. These problems often originated from the necessity to determine the content of toxic elements in relevant matrices with sufficiently low uncertainty, which for legal or medical upper limits exist, and to come up with comparable measurement results within the community of NMIs. The rather phenomenological comparability achieved, which is often based on a consensus value, was only sometimes supported by reference measurements, and was used in the first instance to generate evidence for Calibration and Measurement Capabilities (CMCs) for the investigated measurement problem. This is a very sensible approach to quickly establish measurement comparability within accepted levels of uncertainty and distinctly higher than the uncertainty of primary transfer standards (PTS). A transparent demonstration of the traceability chain of the measurement results down to the SI was not an urgent necessity for the NMIs. Often the very commonly used traceability statement “traceable to {NMI name}” was all the information requested and provided. This practice arose due to the special situation of metrology in chemistry in the very beginning of its existence in formalised form and was fully appropriate for that time because there was simply no acceptance for establishing the underlying PTS as such activities would have drastically restricted addressing the burning societal challenges. Besides this, generating trust of society in the newly formed international body, and supporting worldwide trade were important missions. The list of approved CMCs and the list of Key Comparisons (KCs) and Pilot studies (Ps) clearly reflect the focus of the extensive and versatile international cooperation in the IAWG over its 25 years of existence, with a small part being dedicated to PTSs [6].

It is the responsibility of the NMIs and not less than their right to exist to develop and maintain PTSs and to establish mechanisms for their dissemination. By nature, PTSs from NMIs are national standards with a legal meaning. Other bodies such as companies, public institutes, CRM producers, accredited laboratories, or other technical competent laboratories can in principle also prepare and maintain standards with potentially high technical quality. If, however, PTS can be issued by non-NMIs is still an ongoing debate. The authors are of the opinion that PTSs from non-NMIs have no legal meaning, as they do not guarantee (international) comparability as covered by the CMCs with the underlying measurement capabilities review process. The legal meaning of PTSs here is not restricted to legal metrology, although having implications on it, but it demonstrates the international system of metrology and the fact that only officially appointed institutions such as NMIs and DIs can contribute here via CMCs.

**Fig. 1:**
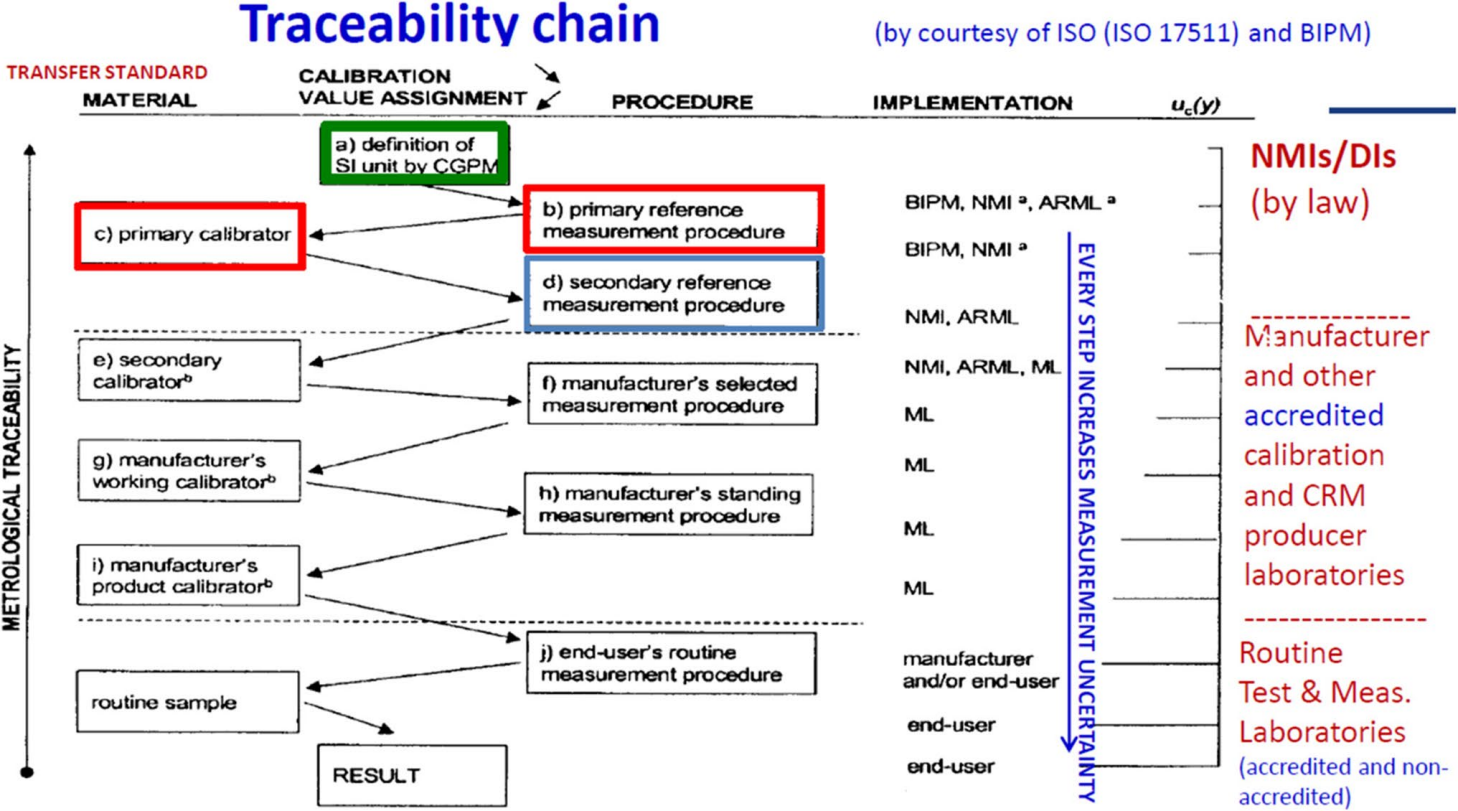
Scheme of a full traceability chain (from [4] reproduced with kind permission from Prof. Robert Kaarls); here, the PTS is named primary calibrator

PTSs, however, are indispensable in metrology in general and in legal measurement systems in particular. For the embodiment of the abstract definition of the SI unit, which is the end point of the traceability chain (Fig. 1), a PTS is needed, often named primary standard or primary calibrator. By definition, the value assignment for this PTS has to be done using a primary measurement method or a primary measurement procedure to obtain a measurement result without relation to a measurement standard for a quantity of the same kind [7].

As in chemistry, there is no universal method of measurement which is independent of the analyte; a variety of primary standards preferably for each chemical identity (element or compound) is needed. This fact is more than just a technical problem; it is the consequence of the essential nature of chemistry and hence of chemical analysis. This high grade of diversification is typical for chemical measurements and — at the same time — a complication that is not to be found in an analogous form for any other (physical) quantity to be measured and that has far-reaching consequences.

The necessity to establish primary standards for all of the relevant types of chemical analytes has the consequence of requiring an extremely large effort for each NMI, as laid out in [8]. Additionally, matrix effects make the practical implementation of metrology in chemistry more difficult. These matrix effects, namely the influence of all components of the measured sample on the analyte signal, cannot be quantitatively predicted by theoretical considerations, but only be experimentally matched. In the case of many practical problems, this requires the certification of matrix-matched standards or matrix certified reference materials (CRMs) beforehand for the calibration of the measurements and for the validation or verification of the measurement procedure.

The certification measurements of such matrix CRMs themselves need to be based on calibration with materials (mostly solutions) whose metrological traceability to the SI is transparent and assured. Therefore, matrix CRMs are not suitable end points of the traceability chain as they depend themselves on primary transfer standards (PTSs).

In chemical analysis, PTSs are materials known for their main analyte content certified by a primary method of measurement. Most easily they are realised as a high-purity material of an element or compound certified by a primary method. This can be by the primary difference method (PDM), i.e., by quantifying the analyte indirectly by subtracting the sum of all possible impurities from the ideal purity of 1 kg·kg^−1^, or by a classical primary direct method of measurement (CPM), i.e., by quantifying the main analyte directly. The activities on primary standards conducted by the IAWG so far are comprehensively compiled by Röthke et al. [9]. It is notable that mainly activities on comparison exercises and studies at least one step further down in the traceability chain than the PTS in the metrological hierarchy are reported, i.e., at the level of calibration solutions that are typically related to (solid) PTS. Apparently, there are only few activities on the underlying solid PTS itself. If these standards are of underlying importance for the metrology and legal measurement systems, the question arises, why only recently and to rather small extent were studies conducted on this topic within the IAWG.

A wide range of technical problems with high social relevance have been addressed over the past years and it is now time to critically review the practice so far for enabling a decision on the future direction. The main emphasis is that comparability in the metrological sense is interpreted as “comparability of measurement results through (SI) traceability”, which is impossible without the underlying PTSs.

In specific cases there are reasons not to emphasize PTSs, especially where large relative measurement uncertainties are involved or can be tolerated, the underlying PTS might not play a significant role in practice. For instance, in trace analysis, relative uncertainties of approximately 10% can be quite tolerable, or in environmental analysis uncertainty from sampling might be as high as 10%. In these cases, it is very unlikely that the content of a calibration solution which is based on the purity statement of a supplier (and gravimetry) has a comparably large uncertainty or is even inaccurate to this order of magnitude. An earlier comparison made by EMPA on commercial calibration solutions, already being one step further down in the traceability chain compared to high-purity materials, indicated inconsistencies in the range of up to a few percent relative [10], which still is significantly below the 10% mentioned above.

On the other hand, it cannot be denied that uncertainties originating from calibration might be relevant for specifications, compliance with limit values or arbitration analysis. Another principal risk comes from undetected correlations, so it cannot be excluded that, e.g., all NMIs use the same wrongly certified starting material, or obtain the same wrongly certified calibration solutions, which would result in severe misinterpretations. A practical example is the case of Rh in CCQM-P46 where, depending on the source of the Rh material, whether obtained as metal or as salt, two distinct levels with a relative difference of ≈ 0.6% were observed among the calibration solutions of the four participating NMIs [11].

There are even more practical reasons to end the current practice and to tackle the problem of establishing and transparently demonstrating PTS by the NMIs. An impressive example are narrow tolerance intervals for components in complex compounds such as high technology materials with unusual stoichiometry such as high-temperature super conductors. Here, trustworthy measurement procedures are required that not only provide small measurement uncertainty but also include for each component a reliable basis for SI traceability.

A further reason to proceed in this direction is the necessity to fully exploit the potential of comparison methods which can only be achieved by keeping the uncertainty in all steps in the traceability chain sufficiently small. This pertains especially to isotope dilution mass spectrometry (IDMS), which can achieve its full measurement power, i.e., as the most important reference method, only when fully transparent SI traceable primary standards are available featuring certified values of the element content with sufficiently low uncertainty, which in turn are used to characterize the isotopic spikes.

In addition to the very practical oriented reasoning for establishing and transparently demonstrating PTS for inorganic chemical measurements, the earlier mentioned general concept of comparability of measurement results through SI traceability is of utmost importance. This requires the existence of PTSs. In any case, a measurement system without PTSs is not meaningful.

This basic principle is also reflected in the normative requirements relevant to accredited calibration and testing laboratories in accordance with ISO/IEC 17025, to ISO 17034:2016 accredited reference material producers and to requirements in other important regulatory documents such as the recent European pharmacopeia [12].

To practically solve this problem, a helpful and directive step forward was the formulation of a “Roadmap for the purity determination of pure metallic elements” [13] as a follow-up from the pilot study CCQM-P149 [14] on the total purity of a zinc material. In this roadmap, three different strategies are elaborated to meet the stringent criteria for certification of high-purity metals as primary transfer standards. One option is the application of CPMs; the other two are related to PDMs as mentioned earlier and discussed below. The formulated roadmap is based on the experience of NMI comparisons over the last 15 years and on the experience of some NMIs [15] with the certification of PTSs. With these proposed strategies, a start has been made to overcome the unsatisfying and important issue that up to now the metrological basis of the NMIs in the field of inorganic chemical measurements was basically not assured. These activities observed so far indicate that the NMIs could be on track for the realisation and transparent demonstration of primary transfer standards if they would extensively follow the outlined strategies. It is also an objective of this paper to provide an accelerated impulse to this important development.

## Primary transfer standards for inorganic chemical measurements: properties, importance, certification, and transparent documentation

To ensure a common understanding, a few fundamental relations need to be discussed.

### Chemical measurements

Our topic is related to chemical measurements, i.e., those for which the identity of the substances matters. The corresponding SI unit is the mole, which accounts for the particulate nature of matter by counting the number of the particles of defined identity. For inorganic chemical analysis, the scope of identity are the elements of the periodic table. Elements are often mixtures of isotopes, which needs to be considered, whereby the “natural” isotopic composition as tabulated [16, 17] is often a good approximation for the actual composition of the element. As isotopic composition can be very accurately measured by mass spectrometry–based methods, a PTS for each isotope is not deemed to be necessary; however, the isotopic composition of a PTS needs to be verified or even certified. This applies as well to IDMS, where the enriched spike solutions must be characterized for its elemental purity and isotopic composition, as explained later in detail.

Chemical measurements are not restricted to the amount of substance (with unit mol). For chemical measurements, the property “mass (with unit kg) of an identified kind of substance” (here of an element or element compound) is used in most cases. Weighing in chemical analysis has been developed from history and is common practice as the concept of a mass comparator (i.e., a balance) is more intuitive and in most cases much more practical than an amount of substance comparator. It is noteworthy that in chemistry the measured mass must always be further specified by the chemical identity of the measured substance. The link between amount of substance and mass of a specified substance is the molar mass, which in itself consists of nuclidic masses multiplied with the isotopic abundances.

When referring to comparability of measurement results in chemistry, there is a fundamental aspect to obey: it is the very nature of chemistry that chemical reactions and compounds are characterised by small integer amount of substance ratios, the so-called stoichiometry. Apparently, this creates interdependency when separate scales for individual chemical compounds and chemical elements are realised.

In other words: the concept of stoichiometry on its own requires that a defined amount of one chemical element is not only compatible with a different amount of the same chemical element, but also that this is compatible to the appropriate amount of a different chemical element. Consequently, comparability in chemical analysis has two aspects. One aspect is comparability between (the scales for) the different compounds, which on its own is not sufficient. The second aspect is comparability to the SI unit, which anchors the scales for the different compounds and provides comparability between different laboratories.

### Primary transfer standards (PTSs) and traceability

Metrological traceability [7] is the “property of a measurement result whereby the result can be related to a reference through a documented unbroken chain of calibrations, each contributing to the measurement uncertainty”. The ultimate reference and end of the traceability chain is, consequently, the abstract definition of the SI unit (with uncertainty zero). This abstract definition must be embodied or materialised in order to have it experimentally accessible for comparison (i.e., for calibration). The term “embodiment” holds especially true for chemistry, as chemistry by nature deals with matter. The best realisation of the transfer between the world of the abstract definition and the experimentally accessible world of materials is the primary transfer standard (PTS). The additional adjective “transfer” is used in this paper to emphasize this special role. A PTS as the best realisation of the abstract definition of the SI unit is consequently not dependent on any other standards of the same quantity and consequently carries the smallest possible uncertainty. A PTS is therefore an element of central importance in the traceability chain and an integral part of traceable measurement results. Reading the traceability chain with decreasing uncertainty in the reverse direction is the dissemination of a measurement unit with increasing uncertainty.

### Material and immaterial aspects of PTS

PTSs carry by nature a material and a non-material aspect. In chemistry, a PTS consists of a high-purity material. In principle, pure materials (chemical elements or chemical compounds with defined stoichiometry) or solutions — if directly certified — can be used. Ideal purity, however, does not exist in real materials. When a sufficiently large sample is analysed with a sufficiently sensitive methods, at least for the elements, almost everything can be found in everything.

In elemental analysis and within the scope of this discussion, the substance is present in elemental form or as a defined compound (when the elemental form is not stable at ambient conditions, e.g., alkaline metals). The content of the main element has to be measured with small uncertainty using a primary classical direct method and finally has to be certified. This is the material aspect which is needed for dissemination in the reverse traceability chain. Usually, dissemination is performed by using primary solutions, which are either directly certified as PTS by means of CPMs or are prepared from the solid PTS as “primary solutions”.

The non-material aspect of the PTS is the abstract value which it embodies. This is an abstract well-defined statement, which consists of a quantity definition, a value, an uncertainty, and a unit. The quantity definition has to include a specific (prescribed) procedure, how the material has to be treated (e.g., certain cleaning procedure) and might include a prescription of the primary method of measurement in order to realise the embodied value (which is the content of the main element).

From the pure concept of counting, one primary transfer standard should actually be sufficient, and all other standards can be linked to it. In chemistry, however, this is impossible because there is no generally applicable measurement principle which could determine all analytes using just one transfer standard. This gets even more complex due to the matrix effect. Consequently, in chemistry, a large number of primary transfer standards, i.e., for all the different compounds, for the different elements and maybe even for the different isotopes, is required.

### Role of matrix and matrix effect

The analyte is always present in a sample of a more or less complex composition (i.e., the matrix). To take the information on the sample into account, the measurement result is usually expressed per mass in the form of amount of substance fraction (in mol kg^−1^) or mass fraction (in kg kg^−1^). The information on the matrix is given per mass, as mass is nonspecific; i.e., the identity of the particles contributing to the mass of the matrix does not matter for the quantity value and the information on the matrix is nonspecific; i.e., their chemical identity is not known. The matrix, *i.e.*, everything in the sample apart from the analyte, can also have a considerable influence on the analyte signal, which is reflected in a variety of strategies for calibration.

The matrix effect implies that the direct comparison of the content of one element in a complex (solid) matrix against the content of the same element in a different matrix, such as a solution, is not straightforward. More abstractly phrased: direct comparison of two rather different quantities (in size and definition, especially concerning the matrix) of the same class is difficult. To mitigate the matrix effect and enable rather easy calibration, the problem of the measurement of element content in a complex (solid) matrix is transformed via decomposition and, if applicable, via matrix separation to the problem of the measurement of this element in a (simple) aqueous solution. This comparison can then easily be achieved against the content of a calibration solution directly prepared from or linked to a PTS.

The usually solid primary transfer standard consequently needs to be dissolved to obtain a solution. The solvent itself could also be classified as a kind of primary transfer standard (preferably) for the (zero) content of the analyte. Thus, in this case, the generated calibration solution is a (usually gravimetric) mixture of two primary transfer standards, and the value for its content is calculated and certified accordingly. When preparing the calibration solution, loss and contamination of the analyte need to be taken into account (preferably by avoiding them). The element content in the solution will also carry a larger uncertainty than the PTS. Thus, the solution is of secondary nature in the traceability hierarchy, but it nevertheless has to be regarded as a “primary solution”, since it is at the highest position among the solutions in the hierarchy. When using CPMs — as discussed later — such as coulometry, the content of a solution can be determined and certified directly as a PTS without requiring preparation data.

Standards derived from PTSs (in inorganic analysis often in the form of calibration solutions) are used for the first step in calibration as defined in the International Vocabulary of Metrology (VIM) [7], i.e., to experimentally determine the relationship between the intended to be measured quantity (the content of a chemical element) and the actually observed quantity (a measurement signal).

## Strategies for implementation of PTSs

### General aspects

As evident above, a whole variety of PTS is required in chemical analysis, and the question comes up, which practical steps for the implementation in inorganic analysis need actually to be taken. Groups to consider range from elements, isotopes, oxidation states, metalorganic compounds to anions in metal salts. The effort for certification of inorganic PTSs is high due to the huge variety and this creates a challenge for each individual NMI as well as the entire community of NMIs. It seems therefore to be advisable to follow a step-wise approach and to balance effort vs. importance and urgency.

Considering the effort, it is preferable to start with those elements which are non-radioactive and solid at standard conditions (may include Hg). Looking at urgency, usually elements with toxicological, ecological, or technical relevance must be considered. Questions on the need of transfer standards for oxidation states (Cr^3+^ and Cr^6+^) and metal–organic speciation are not discussed herein. Concerning inorganic analysis, elements which are gaseous at standard conditions are not considered herein as gas analysis requires specific technical implementations, which is also left for separate discussion. However, the difference strategy as discussed below can be applied to gaseous elements as well as non-gaseous elements, with the advantage that the number of potentially occurring impurities for gaseous elements might be smaller.

Candidate materials should be of a minimum purity as this generally leads to a lower uncertainty on the value of the main element. In addition, the prescribed cleaning and handling procedure might also include criteria for the pre-selection of candidate materials in order to achieve the desired properties of the PTS with the tools available. Finally, the available amount of the candidate material is an important aspect, because once certified, the PTS should be available as long as possible.

For certification of the content value of the main element, the mass or amount of substance of the defined element will be used. Even though the quantity amount of substance content expressed in mol kg^−1^ would be the alternative from a metrological point of view, for practical reasons, the widely accepted quantity used is mass fraction expressed in kg kg^−1^. As explained above, this is simply due to the practical access to mass using a balance and the very often negligible uncertainty for converting between amount of substance and mass by the concept of molar mass.

It is important to note that even though the certified primary transfer standard is by nature a material, it is an embodiment of a quantity value and not an artefact defining the quantity value. This is relevant because it stresses the non-material aspect of the PTS, which also reflects the effort to realise PTSs in general not as artefacts. The non-material aspect of the chemical transfer standard is the procedure for realising this standard, which includes a primary method of measurement, and is in that sense the essential aspect of a PTS. Based on the information contained in the non-material aspect of the PTS, PTSs of the same kind with comparable properties can be certified independently from any available starting material. The resulting new PTS will usually not meet exactly the same quantity value and uncertainty, but the metrological quality of both PTS materials will be comparable within sufficiently small measurement uncertainties. This is important if a specific PTS is exhausted, or for enabling other NMIs to produce such PTSs once the methodology is developed.

### Requirements and consequences derived from the traceability chain

It is possible that an NMI certifies a PTS for a specific chemical element with rather large relative uncertainty of let us assume 0.3% relative. Consequently, for this element, the NMI itself or any other NMI that links to that standard cannot participate in comparison measurements which require smaller target uncertainties than 0.3%. The same reasoning holds for all other measurements based on this particular PTS, such as the certification of matrix CRMs. The relative uncertainty of the applied PTS determines the minimum uncertainty of all subsequent measurements in the same traceability chain.

This clearly demonstrates the close relation between the used PTS and hence the source of traceability on the one hand, and the explicit capability frame in which an NMI can perform certifications, measurements, and calibrations on the other hand. This is also reflected in the statement “source of traceability” which the NMIs are asked for when applying for mutual approval of CMCs [18]. If an NMI refers here to its own capabilities or capabilities of other NMIs, it should not only be transparent which PTS was used to establish the SI traceability but also how its own PTS has been certified. The entry for the PTS should have an additional link to comprehensive transparent documentation of its certification. Currently within the CCQM, there is no such harmonised information platform foreseen. Therefore, each NMI, which certifies PTSs, provides linked secondary standards, or provides linked measurement or calibration services, should provide transparent documentation on all aspects of their PTS. This could be achieved via the webpage of the NMI or a suitable data repository. It is up to each NMI to decide whether this data is available to the other NMIs only, or whether this data is publicly available. Transparency, at least within the NMI community or publicly, seems to be mandatory as a confidence-building measure. Additional peer reviews conducted by regional metrology organisations (RMO), which are partly in place, act along the same line and help to identify hidden errors or shortcomings.

### Liquid and solid form of PTSs

**Fig. 2:**
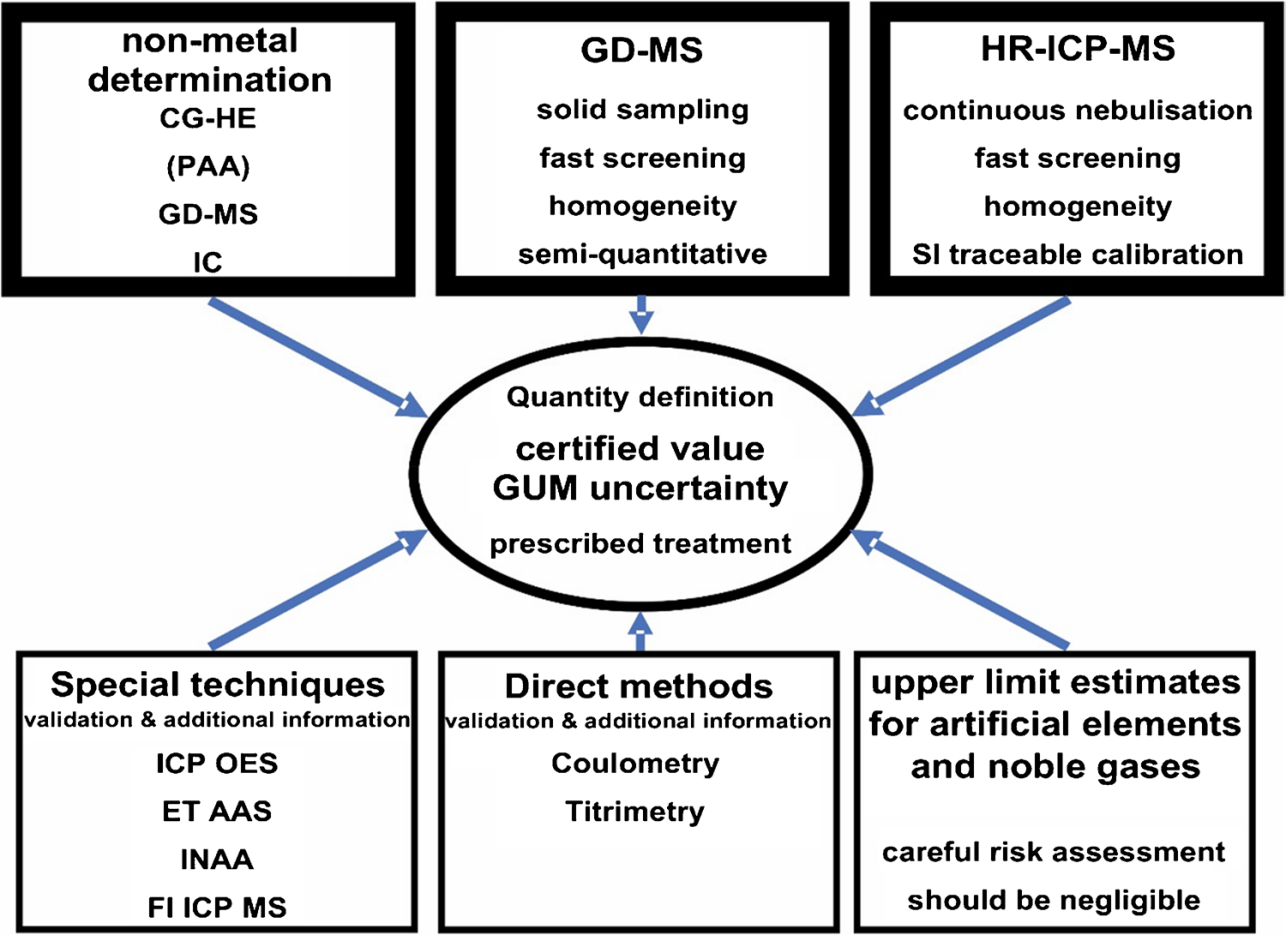
Bouquet of analytical techniques which can be applied to assess the purity of a PTS. Direct methods are listed to be used as CPM; all other analytical techniques can but need not be applied in PDM

Regarding the question in which form (as “pure” solids or solutions) PTSs are produced, a distinction must be made according to the manner of their certification, for which the following explanations serve. When using the PDM, the PTSs are present as solids (or exceptionally as liquid in the case of mercury) in their elemental form. For cases where, due to the high reactivity or other technical reasons, a material of the element itself is not a good choice, binary compounds with well-known stoichiometry could be advantageous (e.g., the use of NaCl instead of Na as metal would be preferred). When applying the PDM concept (for example for copper), the content (usually expressed as mass fraction) of all impurities (i.e., of all elements other than the main element in the candidate material) are determined with suitable methods of measurement (see Fig. 2). In order to assign the certified value for the main element, the sum of the results from the impurity measurements is subtracted from the value of ideal purity, which is 1 kg·kg^−1^. To calculate the uncertainty of the certified value, the uncertainties from all impurity values need to be considered. Due to the fact that no calibrant of the substance type to be certified as PTS (in the example no copper in any form) is used for the certification measurements, the primary character of the method is obvious. Even though most of the individual impurity measurements are made on the dissolved candidate material, properties of the solid starting material (pure copper in the example) are finally certified, and this represents the PTS for that element.

Solutions prepared from the PTS are not a/the PTS in itself; they are secondary standards, derived from the primary standards (the PTS) by the step of dissolution (i.e., digestion and mixing with the solvent). Consequently, the values attributed to the solutions carry by nature a larger relative measurement uncertainty than the PTS itself. In most cases, solutions also have lower shelf lifetime. Nevertheless, these solutions can be called “primary solutions” as they are at the top of the traceability hierarchy of calibration solutions.

While a direct purity comparison of solid materials is very difficult, solutions are in a suitable form for rather easy comparison measurements against other solutions. This enables calibration (after suitable dilution) as well as dissemination of the metrological traceability chain to the field laboratories. Note that, compared to other CPM (such as coulometry), the PDM is not suitable for directly certifying solutions as PTS. It is the purity of a (normally solid) starting material which is determined on the basis of the measurement of all impurity elements in this material.

### Classical primary method (CPM) and primary difference method (PDM)

The advantages of the PDM are obvious: The method is universally applicable to all chemical elements considered here and the concept can also be applied to other inorganic compounds or even metal–organic compounds. The actual measurements are further based on the use of well-tested, validated individual methods having a high analytical selectivity, which especially applies to atomic spectrometry. This prevents gross errors, such as in particular the incorrect assignment of analytical signals (spectral intensities) to analytes that do not belong to those signals. Another advantage is the general possibility to achieve very low uncertainties for the certified value (the mass fraction of the main element in the PTS). To achieve this, it is mostly important to choose suitable candidate materials and to adapt sufficiently sensitive measurement methods for the determination of the impurity elements. This implies that very pure candidate materials with preferably correspondingly low mass fractions of the impurity elements are identified. In this case, very high individual relative measurement uncertainties can be tolerated for the contents of the trace impurities, even when aiming for extremely low uncertainties of the main element content to be certified.

As the relative uncertainties of the measurement methods of the individual trace impurities are usually constant at first approximation (at least in terms of order of magnitude) over wide ranges of values, and these uncertainty contributions are added quadratically, it is easy to show that a significantly higher mass fraction of just one of the impurity analytes leads to a significantly increased uncertainty of the certified mass fraction of the main element.

Unfortunately, non-metallic analytes, especially oxygen, are often a major problem that makes the selection of suitable candidate materials complex, because usually the manufacturers of high-purity materials do not give specifications for these analytes and do not take these analytes into account for their initial purity statement. The purity statement for the mass fraction of the main element from commercial producers of high-purity materials is usually expressed as the “number”(X) of “nines” (N), e.g., “6N”, and often restricted to only “metallic” impurities, e.g., expressed as “m6N”. However, for the selection of a suitable candidate material for a primary transfer standard, total purity, e.g., expressed as “t3N”, matters.

In practice, the metallic purity often is orders of magnitude higher than the total purity. An example is discussed in [19]. A further complication arises from the difference between (variable) surface and (often constant) bulk contents of oxygen. This requires a prescribed relation of volume to surface geometry of the individual material pieces of the PTS, and a prescribed cleaning procedure to be applied immediately prior to weighing, such that the well-defined state is reached for which the certified value applies. Very sophisticated methods, such as photon activation analysis and then removing surface oxygen, can be used to reliably distinguish between oxygen on the surface and in the bulk of the material [20–23]. For the measurement of “gaseous” components in the candidate material, only direct solid sampling methods can be used, such as glow discharge mass spectrometry (GDMS), carrier gas hot extraction (CGHE), or the combustion method for other non-metallic impurities.

For purity characterization, suitable methods preferably having the lowest limits of quantification (LOQs) and smallest measurement uncertainties should be selected. However, fast and multielement methods are preferred, at least for the measurement (maybe for first qualification) of metallic impurities. Impurity contents which are below the LOQ also need to be considered in the calculation of the final result, which by nature involves expert estimates. Especially for high-purity materials, this is often the case. As long as the values are low, it seems to be reasonable and appropriate to apply for each impurity an estimate of (*w*_LOQ_/2 ± *w*_LOQ_/2) for the value and for the uncertainty of the mass fraction. (More sophisticated calculation approaches using rectangular- or beta-distributions [24] are also possible.) The same mathematical treatment applies for upper limit value estimates, which are needed in case of impurities rarely occurring in the specific matrix and which cannot be quantified by measurements due to lack of suitable methods or lack of a suitable calibration base. Frequent candidates for upper limit estimates are the impurities with radioactive (artificial) elements, noble gases, and to some extent halogens. Based on a risk assessment, estimates for upper limit values for these impurities must be made taking the history and the production process of the material into account. The contribution of upper limit estimates to the total purity statement should be small, and wherever possible replaced as soon as possible by experimental data.

It should be noted that when applying this classical concept of symmetrical uncertainties, and by adding a large number of (*w*_LOQ_/2 ± *w*_LOQ_/2) statements of similar size, the combined uncertainty could possibly no longer cover the corresponding quantity value. In practice, however, often one or few individual impurities in the candidate material dominate the impurity statement. The mass fraction of those dominating impurities in turn needs to be measured with a much lower relative uncertainty to avoid larger combined uncertainties of the mass fraction of the main element.

**Fig. 3:**
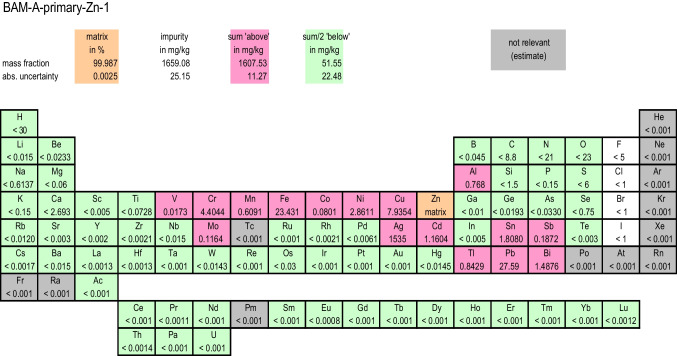
Summary of the results of impurity measurements of BAM-Y014, the PTS for Zn. “sum above” is the sum of all impurity values found above the respective limit of determination or upper limit; “sum below” is the half of the sum of the respective limits of determination for impurities found below the limit of determination or impurities that are below an upper limit value

In addition to the advantage of its great universality and the possibility to achieve very small uncertainties for the mass fraction of the main element, the PDM has also some disadvantages. First and foremost, there is the high effort involved in pre-selecting the candidate material and in determining not less than 82 analytes with suitable and validated/verified analytical methods as displayed in Fig. 3 [13]. Another argument against the use of PDM is that even though the basic requirement for a direct primary measurement method is met, the quantification of the impurities requires calibration standards, which in general are not necessarily fully SI traceable themselves. However, it should be kept in mind that the relative uncertainties of the measurements of the mass fractions of the impurity elements can be given in a very conservative way (e.g., up to 30% relative) without jeopardizing the achievement of very low uncertainties for the determination of the mass fraction of the main component (within, e.g., 0.01%). Compared to the uncertainty of the measured value for each impurity element (of e.g. 30% rel.), an assumed improbably large uncertainty (e.g., 10% relative) on the calibration standard used will only result in a small contribution to the combined uncertainty, due to the quadratic addition. This shows that even large uncertainties on the calibration solutions for the measurement of the impurity elements do not compromise the basic concept of the PDM. However, this requires a high purity of the candidate material for the primary transfer standards.

In contrast to the PDM, CPMs, such as gravimetry, titrimetry, or coulometry, are well suited to certify (element) solutions directly as PTS. Applying CPM, the overall composition of solid candidate materials does not need to be determined because elements not contained in the solutions prepared from them (such as oxygen or other gases) are not important in the certification process. This is a considerable advantage of CPM over PDM, because the measurement of gaseous elements can be considerably difficult, and they require the availability and experience with less common analytical methods.

Although the analyte content of the solution can often be calculated beforehand from the known data for the solid starting material and the gravimetry data, certification by CPM is only based on the measurement of the solution. Even losses of the main element or contaminations with the main element in the preparation process do not matter. Nevertheless, a documentation of the history of the candidate solution (as far as concerning the handling in the certifying NMI) would be favourable with respect to the transparency of the certification process of the final PTS and for increasing its acceptance.

Despite these advantages, CPMs have significant limitations compared to PDMs. The major disadvantage is the limited number of applications, which in addition require specific approaches; hence, CPMs do not allow universal application to all the elements considered here. Another disadvantage is the significantly lower selectivity compared to the measurement methods used in the PDM. This can result in a usual overestimation of the content, potentially causing undetected gross errors (blunders) for the certified value of the PTS. A typical example from gravimetry is precipitation and co-precipitation reactions of elements other than the main element for which a PTS is certified. The supplementary use of (element) specific, usually atomic spectrometric measurement methods, can compensate for this deficiency, but only if all interfering impurities are addressed. It is important to note that the overall uncertainty for the PTS is increased to the extent that such non-specific interferences occur.

A typical example for CPM is titration with EDTA solution. An EDTA solution is produced and calibrated (the titer) against a solution of Zn^2+^ which in itself has been produced from a high-purity (Zn^0^) standard yielding the so-called primary titer, i.e., a standard which actually has to be characterised by PDM. Obviously, a PTS certified by titration with an EDTA solution cannot have a smaller uncertainty than the (Zn^0^) standard. CPMs can also have very specific methodical intricacies, as can be shown for the example of EDTA titration: different metals also react differently with EDTA; different pH values have to be maintained and, in addition, it would have to be demonstrated that all metal EDTA reactions proceed also equally in the low measurement uncertainty range, a range which is usually not investigated. Independent of the practical limitations, both approaches (PDM and CPM) to certify PTS are basically appropriate.

### Quality of PTSs

The question of the required quality of a PTS has not yet been answered. Of course, a PTS must be of high metrological quality; metrological validity supersedes smallest uncertainty. However, it is also clear that measurement uncertainties play a key role because the PTS holds the top position in the traceability chain and thus determines the minimum uncertainties of all subsequent levels, down to the field laboratory. From each level to the next level in the traceability chain, the measurement uncertainty increases, theoretically at least by a factor of ≈ 1.4 (addition of two approximately equal uncertainties). When the target or intended value of the measurement uncertainty of a PTS is assessed, different approaches might be applied. A pragmatic approach, described above, is the consideration of the individual tasks of an NMI and their associated uncertainty requirements. Based on this, the uncertainty of the PTS can be assessed by going up, level by level, in the traceability chain; in the example above, the relative measurement uncertainty of the PTS was 0.3%. On the other hand, a generic approach, in the sense of metrology, could be the consideration of the applied measurement procedures and the selection of the measurement procedure with the highest performance. In elemental analysis, this is IDMS [25, 26], because it achieves smallest measurement uncertainties and thus is the most frequently applied reference procedure.

### The role of isotope dilution mass spectrometry (IDMS)

In IDMS, the ideal internal standard is added to the sample, the so-called spike, which consists of an enriched isotope of the analyte element itself. Before the addition of the spike, the isotope ratio for the analyte in the sample is measured in case relevant differences from its tabulated “natural value” are to be expected. In the spiked and isotopically homogenised sample, only a specific isotope ratio of the analyte element has to be measured and together with the spike data, the weighing data of the added spike and the sample and the molar mass, the analyte amount of substance or the analyte mass fraction in the sample can be calculated. After the mixing of spike and sample, a loss of analyte to a first approximation has no effect on the result, because only isotope ratios are measured, and each subsample represents the same isotope ratio. This permits an extensive analyte-matrix separation, which removes a major part of the sample matrix and therefore most matrix effects and spectral interferences. With thermal ionization mass spectrometry (TIMS), matrix separation is mandatory to enable isotope ratio measurements, and with inductively coupled plasma mass spectrometry (ICP-MS), it is recommended when applying IDMS as a reference method. Thus, IDMS is largely unaffected by analyte loss and by matrix-effects (provided complete equilibration of the added spike has been achieved), leading to the high robustness and ruggedness of the method. Additionally, IDMS intrinsically considers variations in the isotopic composition of the elements which occur in natural samples as well as in technical ones. This is also especially important for the certification of PTSs where the raw materials undergo purification procedures, which might shift the isotopic composition of a PTS outside the natural range [17].

It should be noted here that IDMS is applied as well in a simplified manner, where isotopic variations are not considered, matrix separation is not carried out, and other approximations are made. This is often the case in routine applications (e.g., multi-element analysis), online applications in hyphenated systems (e.g., LA-ICP-MS, HPLC-ICP-MS), and several others (e.g., nanoparticle analysis). In this context, however, we consider only IDMS applications from the viewpoint of a high-quality reference measurement method, more specifically, a primary ratio method of measurement.

IDMS, as any other method, is not per se a primary method of measurement, but it can be applied as such, provided specific requirements such as a completely understood measurement process, negligible corrections, and complete uncertainty budget are fulfilled. IDMS is considered not as a primary direct method for amount of substance measurements leading to the SI without any external reference, but as a primary ratio method for amount of substance measurements, which requires an external reference. The term is defined by CCQM and is provided with explanatory remarks [7, 27, 28]:

“A primary ratio method measures the value of a ratio of an unknown to a standard of the same quantity; its operation must be completely described by a measurement equation.”

Explanatory notes:

1. A primary direct method can be used to make a measurement that is traceable to the SI without the use of an external reference of the same quantity (for example, gravimetry or coulometry).
2. A measurement traceable to the SI can be made using a primary ratio method in combination with a reference of the same quantity that is itself traceable to the SI. However, a method whose operation cannot be completely described and understood cannot be a primary ratio method.
3. A primary direct method can be combined with a primary ratio method to produce measurements that retain their primary qualities (for example, isotope dilution mass spectrometry with a gravimetric assay of the pure spike).

The external reference in IDMS is the isotopically enriched material or spike (single IDMS) or the primary assay or so-called back-spike, which is used to characterize the spike in a so-called reverse IDMS. Combining the reverse IDMS for spike characterization with IDMS for sample analysis leads to the so-called double IDMS experiment. Due to technical and economic reasons, it will not be possible to determine the mass fraction and the isotopic composition of the analyte element in all available spike materials/solutions in a similar way to the PTS, especially when considering that a PDM approach is not applicable to solutions. The more efficient approach is to use PTSs as back-spikes in double IDMS experiments. Allowing the analyst to take advantage of the full potential of IDMS relative measurement uncertainties of 0.1% (in special cases even 0.05%) can be achieved [26]. This, however, requires the determination of the isotopic composition of the PTS. As noted above, PTS can show significant deviations from the natural isotopic composition due to the applied purification procedures (e.g., distillation). Once the isotopic composition of the PTS has been determined, the application of the PTS can be extended to back-spike applications, which offers two major advantages. First, the purity of the PTS can now additionally be calculated as amount of substance content by using the molar mass obtained from the isotopic composition. Second, the PTS can be applied as primary back-spike, which can be used to produce back-spike solutions. The latter can be used to determine the amount of substance content in the spike solution and the purity of the isotopically enriched material, which hardly would be possible or economic via direct analysis as in most cases the available mass of the enriched isotopes is in the milligram range. Thus, spike solutions with SI traceable amount of substance concentrations and/or mass fractions will be obtained. Using these “traceable” spike solutions, the traceability chain in IDMS can be closed and complete uncertainty budgets can be set up, which is not possible without PTSs. When applying multi-collector mass spectrometers, the mass fraction of the back-spike, and thus the purity of the PTS, is one of the major contributors of double IDMS uncertainty budgets. Starting from a relative measurement uncertainty of 10^−3^ (0.1%), which can be achieved by double IDMS, and adding the requirement that the uncertainty of the back-spike should not compromise this uncertainty to maintain the metrological quality of IDMS, we end up with a requirement for the uncertainty of the back-spike of < 10^−3^ (< 0.1%). Considering additionally that the preparation of the back-spike solution from the solid material adds additional uncertainty, it seems reasonable to require a relative uncertainty of 10^−4^ (0.01%) for the purity of the solid material, the PTS. Following this conceptual approach, a universal system could be established building the primary basis for the metrological traceability of the key reference method in elemental analysis, the IDMS approach. This demonstrates the second fundamental importance of a PTS.

## Status and perspectives

As discussed earlier, the PDM seems to be the method of choice in order to certify the materials terminal link of measurement standards for element determination having very high precision of the certified value. The question arises as to what has been achieved so far concerning the analytical methods involved, concerning the technical realisation of this concept, and concerning international comparisons?

Multielement impurity measurements in the liquid phase after (loss free and contamination free) decomposition, based on ICP techniques and on ICP-MS in particular with sufficiently low limits of determination, have been described in detail [19, 29–31]. Analyses applying liquid-based methods is independent of the sample form (powder, geometry) and can usually be calibrated with good SI traceability, even for exotic elements. If needed, especially for dominant impurities, quantification can be performed with small uncertainty.

Determination of non-metallic impurities (O, N, H, C, S) is of special importance, as they are in general difficult to measure and are often the dominating impurity contribution (especially oxygen) with even the complication of a surface and a bulk contribution. By nature, most non-metallic impurities cannot be measured after dissolution. CGHE, in particular, can be applied here and is constantly being developed further [22, 32–34].

Solid sampling methods, which avoid losses and contamination of analytes in a potential decomposition step, are very valuable. GDMS, in particular, is a comprehensive multielement method with high sensitivity, detection limits in the ng/g range, and a linear response over more than 10 orders of magnitude [35] and has been applied for the purity determination of metals. The technique is well established for screening and semi-quantitative analysis [36–39]. When experimentally determined sensitivity factors for defined analyte matrix combinations are lacking, extensive concepts have been developed to estimate the sensitivity factor from other data [35, 40, 41]. A methodology to transfer the versatility and traceability concept of liquid calibration in ICP-MS to GDMS by using liquid doped calibration standards has been performed [39]. GDMS when applied for non-metal analysis requires enhanced ionization efficiency, such as by addition of He to the plasma gas [42, 43] and for the preparation of calibration materials by solid doped powder samples [21]. Depending on the instrumental setup, GDMS requires the sample to be in a specific geometric form (pins or flat samples). All in all, GDMS can play an important role in realising the difference method to certify the materials end of primary standards for element determination [35].

The reliable determination of halogens at trace level is a difficult task. The decomposition step for solution-based methods such as ion chromatography is crucial. Here, nuclear methods such as PAA [20] offer powerful potential. Similarly, NAA and INAA [44] offer powerful potential for the determination of metallic impurities. Nuclear methods are rather easy to calibrate with small uncertainty and good SI traceability; they are not prone to contamination after the activation process; however, they are not applicable for each analyte/matrix combination, and they require a lot of effort and infrastructure.

Finally, for converting the actual measurand, i.e., the mass fraction of the main element to the corresponding amount of substance content requires to know the molar mass of the actual material with sufficiently small uncertainty. Due to the purification process, the molar mass of a high-purity element might differ substantially from the tabulated average isotopic composition [16, 17]. This has been observed for the PTS for Pb, BAM-Y004 [45], which has been also used for Pb isotope measurements in CCQM-K98 [46]. Thus, isotopic measurements — usually based on mass spectrometry — need to be involved in PTS certification.

The difference method has been applied in several cases [15, 29, 47–50]. The whole chain of applying this concept has been particularly demonstrated in [48]. At BAM, a first set of 10 elemental materials has been characterised for total purity and documented in a transparent and accessible way [47]. At PTB, meteorological calibration solutions have been provided and made available to a producer of commercial calibration solutions. By providing unknown samples from PTB, the producer was tested for its ability to successfully link secondary calibration solutions to the primary calibration solutions. Even though the quality of the resulting secondary calibration solutions was thought to be higher than the existing products, they were not used for marketing and/or selling those products, until the majority of elemental solutions could be offered in this quality commercially. This is understandable but creates an all or nothing situation.

Whereas Röthke et al. [9] summarise the work/comparisons on primary calibration solutions, due to effort and complexity, only very few metrological comparisons [14] have been applied on characterisation of the underlying solid materials. Concerning the evaluation of comparisons of total purity analysis by the difference method, it must be kept in mind that not only the analytical performance of the total purity determination matters, but also their performance for the individual impurities matter, as wrong results for different impurities can mask each other.

To conclude, the existence of materials terminal link in form of (solid) primary standards for element determination is indispensable for a working measurement system. Their certification is difficult due to effort and complexity needed and therefore has largely been put on hold by the metrological community in order to give way to establishing comparability for urgent measurement problems in a phenomenological way.

In order to share the total workload, which would overburden each individual NMI/DI, and to have the chance to achieve the goal with metrological satisfaction, joint effort and shared work is advisable. Learning from the thermometry community, where individual NMIs are specialised and maintain only few dedicated fixed-point cells rather than the whole selection of the international temperature scale, individual NMIs might be specialised for individual elements to realize the corresponding primary standards for element determination. Another approach would be to share the work according to the measurement techniques, e.g., one element is characterised in a group of NMIs with different competencies, e.g., in sample processing and packing, in screening and semiquantitative analysis, in quantitative analysis, in non-metal determination, in nuclear analysis, or in isotope analysis.

This approach would require huge coordination work, an official umbrella or framework, and the need to sort out questions on certificate responsibility. Are we prepared for this, and if not, what is the alternative?

## Data Availability

Not applicable.
